# Effectiveness of stationary cycling with electromyographic biofeedback on neuromuscular control and function in individuals with knee osteoarthritis: a feasibility study

**DOI:** 10.1186/s12891-025-08794-7

**Published:** 2025-06-03

**Authors:** Chia-Ming Chang, Huynh Chung, Horng-Chaung Hsu, Li-Tzu Chen, Ruei-Yi Lin, Hsiu-Chen Lin

**Affiliations:** 1 Department of Physical Therapy & Graduate Institute of Rehabilitation Science, China Medical University, Taichung, Taiwan; 2 Department of Orthopedics, China Medical University Hospital, Taichung, Taiwan; 3 Department of Physical Medicine and Rehabilitation, Asia University Hospital, Asia University, Taichung, Taiwan

**Keywords:** Osteoarthritis, Exercise, Biofeedback, Vastus medialis, Neuromuscular control

## Abstract

**Background:**

To evaluate the effects of stationary cycling with electromyographic (EMG) biofeedback on neuromuscular control and function in individuals with knee osteoarthritis (OA).

**Methods:**

Fourteen knee OA patients were randomized into two groups: cycling with EMG biofeedback of the vastus medialis (EBF group) and cycling without biofeedback (Cycling group). Both groups underwent a six-week cycling program, 30 min per session, twice a week. Outcomes were measured at baseline and post-training. Knee pain and function were assessed using the Visual Analog Scale and the Knee Injury and Osteoarthritis Outcome Score (KOOS). Quadriceps strength and endurance were evaluated with an isokinetic machine, and muscle activation was recorded using a wireless EMG system. A two-way mixed-model analysis of variance (ANOVA) was used to assess the differences between sides and groups. For outcomes that were not normally distributed, the non-parametric Mann-Whitney U test was performed.

**Results:**

Both groups showed improvements in knee pain and function, with no significant differences between groups. Muscle strength remained largely unchanged, but the endurance was improved on the contralateral side (*p* = 0.038, η^2^ = 0.312). Larger muscle activations were observed at various time points during the 30-minute cycling test in the EBF group with significant increases in muscle recruitment in the vastus medialis (p = < 0.001 ~ 0.042, η^2^ = 0.482 ~ 0.996), rectus femoris (*p* = 0.003 ~ 0.040, η^2^ = 0.493 ~ 0.859) and vastus lateralis (*p* = 0.002 ~ 0.036, η^2^ = 0.534 ~ 0.875).

**Conclusions:**

A six-week cycling program can alleviate pain and enhance knee function. EMG biofeedback of the vastus medialis significantly changed the neuromuscular control in the rectus femoris and vastus lateralis during cycling. Therefore, this study demonstrated that stationary cycling combined with electromyographic biofeedback training can enhance neuromuscular control of the quadriceps and potentially improve functional performance in patients with OA.

**Trial registration:**

ClinicalTrials.gov identifier, NCT03484910 (registered at March 31st, 2018).

## Introduction

For older persons, knee joint osteoarthritis (OA) is a leading cause of chronic disability [1]. The symptoms of knee OA are diverse, which include pain, muscle weakness, joint stiffness, swelling, instability, and also functional impairments [2]. The functional limitations of this population majorly involved in the activities of daily living (ADLs), such as walking, climbing stairs, transferring, and bathing [3, 4], and the level of these impairments are related to the severity of knee joint pain [5] and muscle weakness [6–8]. 

According to the clinical intervention guidelines for knee OA, the non-pharmacological method should be recommended first [9, 10]. In other words, designing a structured exercise training program for this population should be applied firstly as the core intervention [11]. Considering that muscle weakness is one of the major impairments for these patients, strengthening exercise was usually the primary part of this exercise training program for increasing muscle power and endurance [10, 11], additionally releasing symptoms, and improving function [12, 13]. The enhancement of muscle strength, physical function, quality of life, and relieving knee pain in people with knee OA can be observed after strengthening exercises either using the isometric exercise [14] or high-intensity strength training [13, 15, 16]. Previous studies in the symptomatic knee OA population showed that the increased cross-sectional area of vastus medialis (VM) was associated with reduced knee pain, decreased cartilage loss, and the risk of knee replacement [17]. Therefore, an exercise that focuses on the selective strengthening of the VM may provide clinical efficacy for this population.

Cycling, as an aerobic exercise and a functional activity, allows people to work on muscle strength and mobility in the lower extremities while exerting lighter loads on the joints compared to weight-bearing activities, such as walking or jogging [18–20]. For these reasons, cycling on a stationary bike, which can provide adjustable resistance during the exercise, maybe a choice for treating people with knee OA [21–23]. Additionally, cycling can be used as a group-based exercise in the community, which can train several patients at the same time by a few instructors or therapists and thus assist in decreasing the cost of the treatment. Electromyographic (EMG) biofeedback, as a method to improve the awareness of the selected muscle activation, has been included in the interventions for people with knee OA and demonstrated some benefits in pain relief, muscle strength, and function [24–27]. The maximum voluntary isometric contraction (MVIC) strength of the knee extensor, i.e., quadriceps, could be enhanced after 8-week isometric training with the EMG biofeedback in VM [26]. An increasing trend has been demonstrated in quadriceps strength after five-week EMG biofeedback isometric contraction training in VM and rectus femoris (RF) [25]. However, after a three-week training, no significant additive effects of EMG-biofeedback in VM and vastus lateralis (VL) to the regular strengthening exercise program, including isometric contraction and close and open kinetic chain exercise within terminal range [24]. Similar results were also found for comparing with a six-week traditional quadriceps strengthening training, which exploring the effects on the neuromuscular control (firing timing of the muscles) during up and down stair [27]. For the current evidence, the treatment effect of EMG biofeedback training would vary for different exercises in the OA knee population.

Previous studies have proved that knee joint training by movement exercise within a limited range with EMG biofeedback in VM and isometric contraction could have similar improvements in the quadriceps strength and better VM activation timing and functional outcome compared with the traditional strengthening exercise. However, most of these training with EMG biofeedback were performed in static conditions, i.e., isometric contractions. Scared study have discussed the application of EMG biofeedback in specified muscle activation during dynamic exercises in training knee OA patients, either its effects. Therefore, this study aimed to investigate whether cycling exercises incorporated with EMG biofeedback in VM could improve the knee joint function for the knee OA population, including quadriceps activation, strength and endurance, knee pain, and self-reported functional score.

We hypothesize that a 6-week stationary cycling training program integrated with EMG biofeedback can be effective for patients with knee OA in reducing pain measured by the visual analog scale (VAS), improving functional performance as assessed by the knee injury and osteoarthritis outcome score (KOOS), enhancing quadriceps strength and endurance, and increasing the myoelectric activity of the VM during cycling exercise.

## Methods

### Study design

This research was a single-blinded, randomized controlled trial study. The participants were enrolled in the Department of Orthopaedics in the China Medical University Hospital. They were fully informed and signed the consent form before enrollment. After enrolling and completing the baseline assessments, the participants were randomly allocated into the EMG biofeedback group (EBF group) or control group (cycling group), and they were blinded to which group they were located.

### Participants

The inclusion criteria were those older than forty-five years old [28] and had tibiofemoral joint OA in either one or both knees. The severity of OA was between grades 1 to 3 of the Kellgren-Lawrence score, which a physician verified with radiograph images. All participants must have been experiencing pain most of the days of the week for at least the previous six months.

In addition, those who would be excluded if they (1) had been diagnosed with OA in other joints of lower extremities, like hip or ankle joints, (2) had a lower extremity joint replacement, (3) had a knee joint arthroscopic surgery or intra-articular injections within three months prior to assessment, (4) had systemic inflammatory arthritis such as rheumatoid or psoriatic arthritis, (5) had lower back pain that referred to the lower limbs, and (6) had any cardiovascular disease or other risk factor which precluded participation in aerobic exercise.

### Equipment

**Fig. 1:**
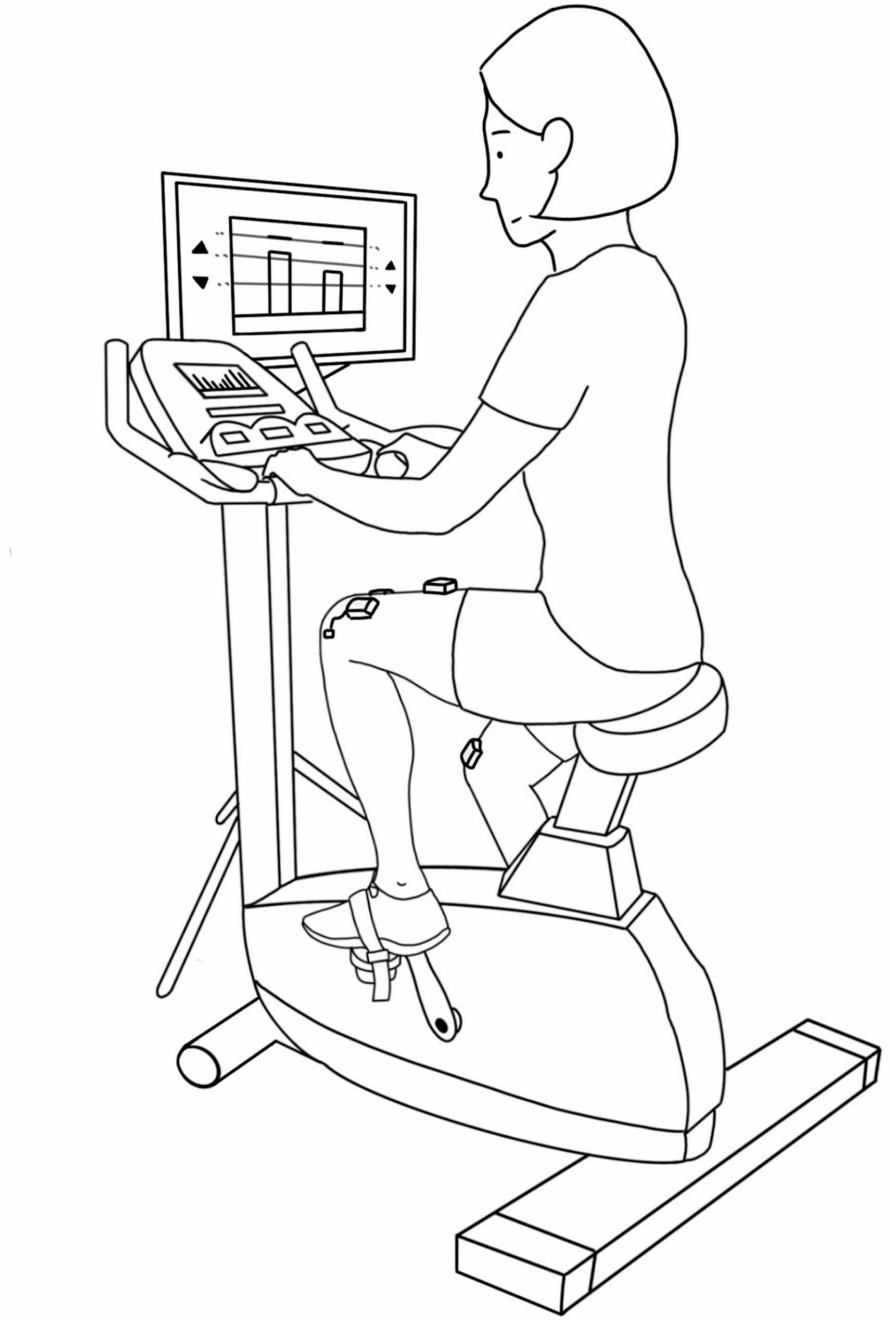
Experimental setup during an EMG biofeedback cycling session

Six wireless surface EMG smart sensors, the Trigno™ Research + system (Delsys, USA) were used to collect the myoelectric signals from bilateral quadriceps, including VM, RF and VL. The VM sensor was located at 3 cm medially and 4 cm superiorly from the superomedial angle of the patella, the VL sensor was 3 to 5 cm above and 6 cm laterally from the superior edge of the patella [29], and the RF sensor was placed on the mid-point between the anterior superior iliac spine and the center of the patella and aligned with it [30]. The VM and VL sensors were aligned with 55 and 15 degree angles to the femur, respectively [29]. Quadriceps muscle strength and endurance on both sides were assessed by an isokinetic dynamometer (Biodex system 3, Biodex Medical System Inc., USA). One upright exercise bike (Cycle Ergometer Matrix U1X, Matrix Fitness, Taiwan) was used for cycling testing and exercise. A screen was placed in front of the exercise bike to show the activity level of bilateral VM in the EBF group. The experimental setup is shown in Fig. 1.

### Interventions

Each patient attended a 6-week cycling program, with two sessions per week. The position on the bike was based on a standard method [31]. The seat height was adjusted to obtain 30° of knee flexion when the crank was at the bottom position (crank angle of 180°) [32]. The 90° angle between the trunk and the thigh was also required by adjusting the position of the handlebars for each participant.

The warm-up procedure was completed firstly on the ergometer bike with the least workload (16 W) for 3 min. Then, during the cycling exercise, the participants were instructed to maintain a workload of 64 W for males and 32 W for females, and the cadence of 60 revolutions per minute (RPM) showed on the bike screen. In the EFB group, the root mean square (RMS) values of the VM activities of both sides were displayed on an extra screen in front of the bike for immediate visual feedback. During the session, each participant was instructed to cycle and make the VM activation bars of the bilateral side as high as possible while keeping the cadence and workload unchanged, as mentioned above. In the cycling group, the participants cycled using the same protocol but without EMG biofeedback. During the cycling session, the participants tried to keep their position on the seat and avoided standing while cycling. The intervention could be stopped if the patient felt uncomfortable. After 30 min of cycling, a cool-down exercise that includes stretching the quadriceps, hamstrings, hip flexors, ankle plantar flexors, and upper extremity muscle groups gently was performed. The training programs and participants’ responses to the exercises, such as heart rate, were monitored by certified physical therapists.

### Outcome measurements

The participants were evaluated before (baseline) and after the six-week intervention by the same investigator. A visual analog scale (VAS) was used to assess the intensity of knee pain and the knee injury and osteoarthritis outcome score (KOOS) Chinese version, which contains pain, symptoms, sports, and recreational function, activities of daily living (ADL), and knee-related quality of life (QoL) subdomains, for functional impairments [33]. The score of each subdomain was recorded except sports, because most of the patients in this study did not perform sport activities.

The Biodex isokinetic dynamometer was used to evaluate the muscle strength and endurance. The maximum isometric quadriceps contraction at 60° of knee flexion and isokinetic knee extension/flexion contraction under 60°/s for five repetitions were used to measure the muscle strength. Further, the muscle endurance was assessed by the twenty-five repeated isokinetic extension/flexion contraction under 180°/s. To understand the muscle activation pattern during low-loading cycling exercise, the EMG signals of VL, VM, and RF were collected for first 10 s every five minutes with a sampling rate of 1,000 Hz during another 30-minute cycling exercise test with the lowest loading, 16 W.

### Data analysis

The peak torques during isometric (TQ_MVC) and isokinetic (TQ) muscle strength tests were recorded and further normalized by the patient’s body weight (BW) as TQ_MVC/BW and TQ/BW. For the muscle endurance testing, the work fatigue in percentage was calculated, and high percentage means better endurance. The EMG amplitude during the isometric test was collected simultaneously as the maximum voluntary contraction (MVC) for normalizing the EMG signals. All EMG signals were processed: 20–400 Hz band pass filter, rectification, linear envelope by 10 Hz low pass filter, and then normalized with the MVC. The maximum of the processed EMG signals would be recorded during the low-load cycling exercise every five minutes. In order to focus on the change by the training, the mean differences before and after the intervention of all outcomes, including functional score, muscle strength, endurance, and EMG activation, were further calculated for comparisons.

### Statistical analysis

The Kolmogorov-Smirnov test evaluated the normality of all variables. If the distribution of the variables fit with the normal distribution, the independent t-test was used to compare the differences in demographic, baseline, and change in functional outcome variables data (VAS and KOOS) between groups. The two-way mixed model analysis of variance (ANOVA) was used to assess the main effects of sides (affected and contralateral) and groups (EBF and cycling) and their interactions. Otherwise, the non-parametric Mann-Whitney U test would be performed. For the categorical variables in demographics, the chi-square test was used to compare between groups. All tests were performed by the SPSS (SPSS Inc., Chicago, IL, USA) statistical program, and the significant level was set at 0.05. Any participants who discontinued intervention or loss to follow-up would be excluded from all statistical analyses.

## Results

**Fig. 2:**
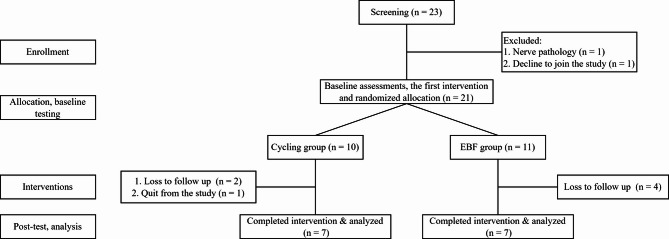
The flowchart of this study

Twenty-three patients were referred to this study from our hospital’s outpatient department of orthopedics. Participant enrollment and following procedures of the study are shown in Fig. 2. After the enrollment screening, one patient was excluded from the study because of the nerve pathology in the lower extremity, and one patient declined to join our study. Twenty-one patients finished the baseline assessment. After that, they were randomly allocated into two groups, ten in the cycling and eleven in the EBF groups. During the intervention, six patients were lost to follow-up: two in the cycling group, four in the EBF group, and another in the cycling group withdrew from the study. Finally, fourteen OA knee patients, seven for each group, completed the 6-week exercise programs and the post-tests. The demographic data of these patients was presented in Table 1, and there were no significant differences between groups.

**Table 1:** The demography data of two groups

	Cycling (*n* = 7)	EBF (*n* = 7)	95% confidence interval	*p*-value
Age (years)	62.08 ± 7.28	64.21 ± 7.33	-10.62 ~ 6.39	0.598
Body height (cm)	161.79 ± 7.19	161.29 ± 7.11	-7.83 ~ 8.83	0.898
Body weight (kg)	65.67 ± 9.83	62.43 ± 8.62	-13.32 ~ 8.38	0.629
BMI (kg/m^2^)	25.07 ± 3.72	24.07 ± 3.13	-5.40 ~ 2.99	0.527
Gender (F: M)	5:2	4:3		0.500 ^a^
Affected side (R: L)	5:2	3:4		0.296 ^a^

^a^ Results from the chi-square test

EBF: EMG biofeedback

BMI: Body mass index

The scores of the pain scale and pain subdomain of the functional questionnaire did not demonstrate the normal distribution, so the Mann-Whitney U test was used for their comparisons. The baseline in the pain scale and functional questionnaire did not show any significant differences between groups. After a 6-week intervention, all patients in this study presented reduced pain and improved self-reported function. The cycling group had a more considerable amount of improvement in the score of pain, symptoms, and ADL, but they did not reach a statistical significance (Table 2).

**Table 2:** Changes in pain and functional scores before and after 6-week training in two groups

Variables		Time point	Cycling (*n* = 7)	EBF (*n* = 7)	95% confidence interval	*p*-value (between group)
VAS		Baseline	3.80 ± 3.07	2.77 ± 1.53	-1.92 ~ 3.97	0.449
		Post-test	2.40 ± 2.26	2.29 ± 1.76		
		Change	-1.40 ± 2.63	-0.49 ± 2.63	-3.98 ~ 2.15	0.620 ^b^
KOOS	Symptoms	Baseline	73.98 ± 13.94	71.43 ± 14.87	-14.23 ~ 19.34	0.746
		Post-test	86.22 ± 9.55	75.51 ± 17.43		
		Change	12.24 ± 7.95	4.08 ± 14.50	-5.45 ~ 21.78	0.462 ^b^
	Pain	Baseline	83.73 ± 12.47	77.48 ± 9.98	-6.90 ~ 19.34	0.321
		Post-test	93.25 ± 7.32	80.56 ± 12.63		
		Change	9.52 ± 10.50	3.08 ± 13.25	-7.47 ~ 20.37	0.333
	ADL	Baseline	86.13 ± 21.89	84.66 ± 11.13	-18.75 ~ 21.70	0.620 ^b^
		Post-test	94.54 ± 3.37	87.39 ± 16.11		
		Change	8.40 ± 19.83	2.73 ± 13.08	-13.89 ~ 25.24	0.651 ^b^
	QoL	Baseline	56.25 ± 21.04	44.64 ± 15.06	-9.70 ~ 32.92	0.258
		Post-test	60.71 ± 18.30	49.11 ± 22.94		
		Change	4.46 ± 10.50	4.46 ± 18.30	-17.18 ~ 17.18	1.000

^b^ Results from the Mann-Whitney U test

EBF: EMG biofeedback

VAS: Visual analog scale

KOOS: Knee injury and osteoarthritis outcome score

ADL: Activity of daily life

QoL: Quality of life

Table 3 shows the muscle strength and endurance changes after the training program. Similar to the functional outcomes, the muscle strength showed no significant differences between groups or sides, and neither any significant interactions at all time points. Both isometric and isokinetic strength showed increased trends in the cycling and EBF groups, except the contralateral side in the EBF group had a slight decrease. For the muscle endurance, there was the main effect (*p* = 0.038, η^2^ = 0.312) between bilateral sides; the affected side demonstrated more positive changes after training, but the contralateral side in the EBF group showed a negative change.

**Table 3:** Changes in muscle strength and muscle endurance performance before and after 6-week training in two groups

Variables	Time point	Cycling (*n* = 7)			EBF (*n* = 7)			*p*-value (η^2^)		
		Affected	Contralateral		Affected	Contralateral		Group	Side	Interaction
Isometric TQ_MVC/BW (Nm/kg)	Baseline	1.42 ± 0.62	1.53 ± 0.47		1.29 ± 0.43	1.45 ± 0.54		0.694 (0.013)	0.290 (0.093)	0.862 (0.003)
	Post-test	1.50 ± 0.63	1.58 ± 0.48		1.49 ± 0.58	1.30 ± 0.31				
	Change	0.09 ± 0.46	0.05 ± 0.44		0.20 ± 0.38	-0.15 ± 0.44		0.804 (0.005)	0.187 (0.014)	0.279 (0.097)
Isokinetic (60°/s) TQ/BW (Nm/kg)	Baseline	1.25 ± 0.53	1.37 ± 0.62		1.18 ± 0.49	1.28 ± 0.34		0.762 (0.006)	0.091 (0.043)	0.826 (0.020)
	Post-test	1.44 ± 0.54	1.49 ± 0.67		1.31 ± 0.61	1.39 ± 0.49				
	Change	0.18 ± 0.23	0.12 ± 0.13		0.12 ± 0.29	0.11 ± 0.28		0.076 (0.788)	0.544 (0.475)	0.250 (0.626)
Isokinetic (180°/s) Work fatigue (%)	Baseline	16.86 ± 16.32	19.31 ± 12.39		13.57 ± 20.88	22.14 ± 7.07		0.975 (< 0.001)	0.182 (0.143)	0.446 (0.049)
	Post-test	26.15 ± 8.61	24.30 ± 8.07		20.58 ± 8.66	17.35 ± 9.82				
	Change	9.29 ± 13.32	4.99 ± 13.39		7.02 ± 22.72	-7.27 ± 11.78		0.352 (0.072)	0.038* (0.312)	0.234 (0.116)

* *p* < 0.05

EBF: EMG biofeedback

TQ: Peak torques

MVC: Isometric TQ

BW: Body weight

**Fig. 3:**
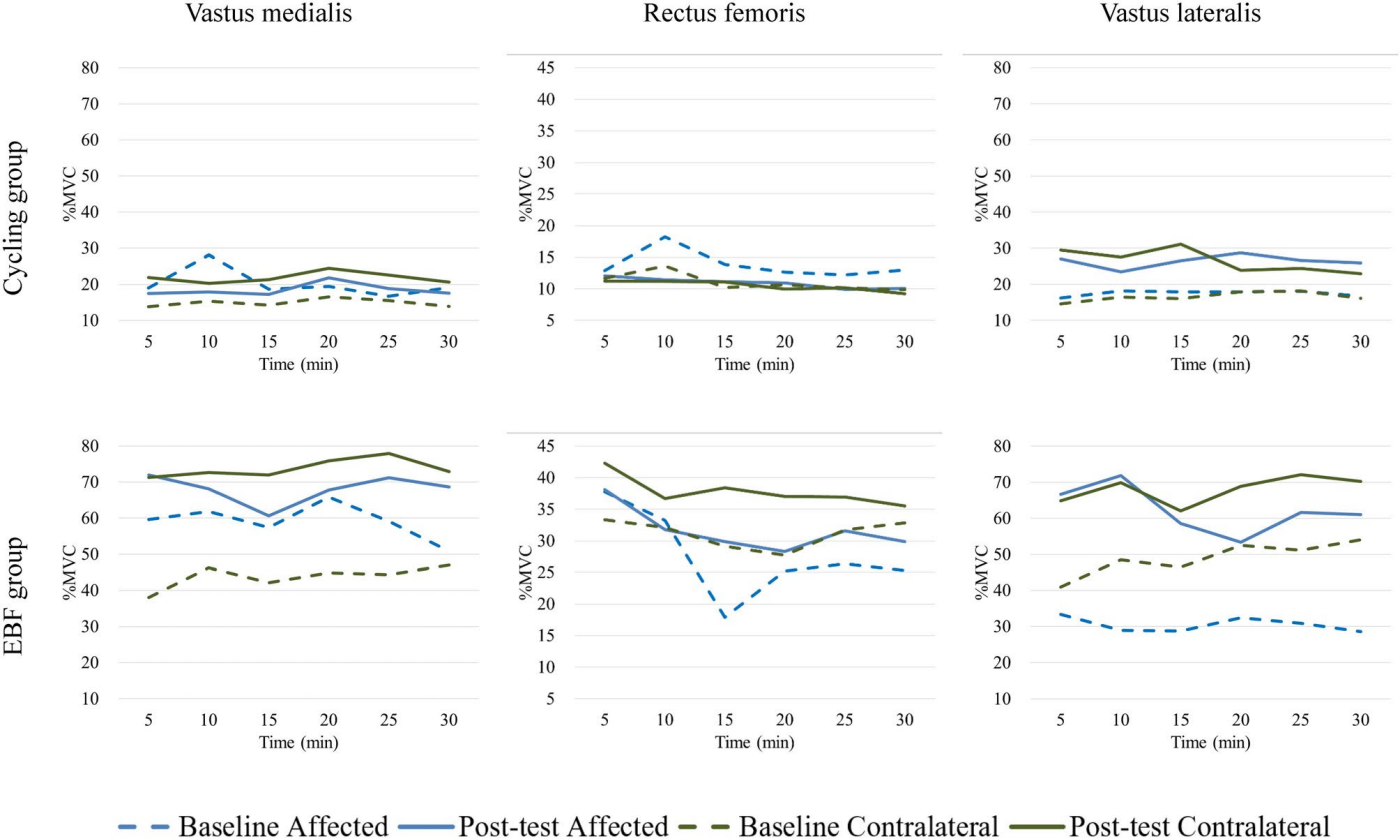
Average of muscle activation level among quadriceps muscles between groups during cycling

The maximum amplitude of EMG between groups and sides during the 30-minute cycling test was presented in Fig. 3. In the cycling group, slightly increased maximum recruitment of bilateral VL and contralateral VM could be observed, but that of other muscles showed little changes or even decreased. Contrarily, all three muscles in the EBF group demonstrated more prominent activation after training, and the increases were much greater than in the cycling group. The statistical comparisons of changes in maximum EMG amplitude (Table 4) showed the group’s main effect, and the EBF group demonstrated significant increases in muscle activation for the quadriceps muscle at different time points (VM: 20 min [*p* = 0.048, η^2^ = 0.450], RF: 30 min [*p* = 0.046, η^2^ = 0.461], VL: 15–20 min [*p* = 0.032 & 0.025, η^2^ = 0.558 & 0.603]). In addition, RF showed the main side effects at the beginning (*p* = 0.048, η^2^ = 0.452) and the end (*p* ≤ 0.005, η^2^ = 0.815 & 0.985), and VL presents the main side effect almost during the whole test (*p* = 0.013 ~ 0.044, η^2^ = 0.486 ~ 0.707), except for the last five minutes. Several interactions were recognized in all muscles, with favoring the increases in either side of the EBF group.

**Table 4:** The mean difference after 6-week training in the maximum EMG amplitude (% MVC) during the 30-minute cycling test in two groups

Muscle		Cycling (*n* = 7)		EBF (*n* = 7)		*p*-value (η^2^)		
Group								
Time		Affected	Contralateral	Affected	Contralateral	Group	Side	Interaction
Vastus medialis	5	-1.56 ± 14.34	8.08 ± 14.10	12.35 ± 38.33	33.21 ± 35.65	0.161 (0.155)	0.245 (0.072)	0.042* (0.482)
	10	-10.18 ± 29.47	4.89 ± 16.66	6.31 ± 42.31	26.40 ± 36.81	0.106 (0.257)	0.331 (0.031)	0.010* (0.734)
	15	-1.38 ± 12.30	7.09 ± 14.32	3.31 ± 42.94	29.83 ± 45.93	0.063 (0.387)	0.254 (0.066)	0.083 (0.318)
	20	2.41 ± 17.22	7.91 ± 16.17	1.96 ± 43.05	30.98 ± 37.51	0.048* (0.450)	0.283 (0.050)	0.155 (0.164)
	25	2.15 ± 12.21	7.03 ± 16.58	12.10 ± 39.05	33.50 ± 42.91	0.106 (0.256)	0.276 (0.054)	0.131 (0.204)
	30	-1.50 ± 14.86	6.69 ± 13.86	17.77 ± 39.59	25.90 ± 43.68	0.119 (0.226)	0.099 (0.272)	< 0.001* (0.996)
Rectus femoris	5	-0.83 ± 3.01	-0.41 ± 4.37	0.30 ± 24.14	8.94 ± 14.98	0.081 (0.323)	0.048* (0.452)	0.040* (0.493)
	10	-6.84 ± 11.40	-2.45 ± 9.89	-1.44 ± 17.76	4.57 ± 16.15	0.079 (0.329)	0.101 (0.269)	0.003* (0.859)
	15	-2.74 ± 4.20	0.86 ± 3.87	1.98 ± 16.97	9.25 ± 16.47	0.104 (0.260)	0.176 (0.135)	0.024* (0.599)
	20	-1.71 ± 6.01	-0.86 ± 2.91	3.09 ± 16.13	9.29 ± 13.95	0.164 (0.150)	0.056 (0.417)	0.029* (0.559)
	25	-2.26 ± 4.01	0.01 ± 2.74	5.22 ± 14.47	5.22 ± 26.90	0.056 (0.417)	0.005* (0.816)	0.005* (0.815)
	30	-2.89 ± 4.14	-0.63 ± 2.55	4.55 ± 13.36	2.48 ± 26.08	0.046* (0.461)	< 0.001* (0.985)	0.015* (0.677)
Vastus lateralis	5	12.41 ± 15.59	12.60 ± 12.58	33.36 ± 45.58	23.91 ± 32.90	0.115 (0.258)	0.013* (0.707)	0.014* (0.696)
	10	8.22 ± 9.53	9.78 ± 12.44	42.93 ± 74.37	21.41 ± 46.02	0.098 (0.298)	0.041* (0.509)	0.054 (0.446)
	15	9.91 ± 13.28	12.66 ± 14.40	29.86 ± 63.58	15.58 ± 44.38	0.032* (0.558)	0.017* (0.672)	0.036* (0.534)
	20	12.56 ± 12.55	3.30 ± 17.53	17.90 ± 51.11	16.38 ± 44.85	0.025* (0.603)	0.020* (0.642)	0.011* (0.738)
	25	9.81 ± 15.65	3.63 ± 18.05	30.74 ± 49.39	20.95 ± 49.62	0.092 (0.314)	0.044* (0.486)	0.002* (0.875)
	30	10.58 ± 15.48	4.57 ± 19.11	32.48 ± 49.28	16.20 ± 55.35	0.063 (0.408)	0.082 (0.344)	0.019* (0.657)

* *p* < 0.05

EBF: EMG biofeedback

## Discussion

In this study, we have implemented the EMG biofeedback of the VM into the stationary cycling exercise and tried to evaluate the feasibility of this EMG biofeedback exercise program for VM strengthening in the knee OA population by comparing the outcomes in the neuromuscular control, muscle function, and functional performance with no EMG biofeedback intervention. This study showed that adding EMG biofeedback may not influence the training effect in functional outcomes, muscle strength, and muscle endurance but could enhance the neuromuscular control of quadriceps.

In the results of our study, the cycling exercise could help to relieve pain (VAS and KOOS-Pain in Table 2), and the changes in the pain scores did not present a statistical difference between groups (VAS: *p* = 0.620; KOOS-Pain: *p* = 0.333). The changes in pain scores after twelve-week cycling training are from 0.21 to 2.29 for VAS and 4.8 ~ 20.0 for KOOS-pain [21, 34], and our results are within the ranges in previous study (VAS: cycling = -1.40 ± 2.63, EBF = -0.49 ± 2.63, *p* = 0.620; KOOS-Pain: cycling = 9.52 ± 10.50, EBF = 3.08 ± 13.25, *p* = 0.333). A previous study demonstrated that isometric contraction training combined with EMG biofeedback for 2 months led to a decrease in the VAS score but showed no improvement in the pain domain of the functional score [35]. In comparison, our study did not find a significant difference in VAS scores between groups. This may be attributed to the lower baseline VAS scores of participants in our study (Table 2), which could have limited the potential for further pain relief in the EBF group. With clinical considerations, the average changes in pain score did not reach the minimal clinically important difference (MCID), 1.6 for VAS [36] and 15.4 for KOOS-Pain [33] (Table 2). In detail, three people in the cycling group (42.86%) and one in the EBF group (14.29%) demonstrated clinical pain relief on the VAS scale, changed over the MCID, and one in each group worsened. For the KOOS-pain subdomain, one person in the cycling group and two in the EBF group presented a decreased frequency of pain, and just one in the cycling group showed more frequent pain. It may reflect that the stationary cycling training could relieve knee pain, and embedded with EMG biofeedback may be beneficial for lowering the occurrent rate of pain.

The functional results in the current study showed that the knee function in both groups was improved in symptoms, ADLs, and QoL categories, and also did not present a difference between groups (Table 2). Similar results were demonstrated in SA Raeissadat, SM Rayegani, L Sedighipour, Z Bossaghzade, MH Abdollahzadeh, R Nikray and F Mollayi [35], which found no significant differences between isometric training with and without EMG biofeedback after two months of intervention. Compared to the improving range of KOOS after twelve-week cycling training (symptoms: 1.3 ~ 11.8; ADL − 0.1 ~ 23.9; QoL 3.4 ~ 25) [21, 34], our results are similar to theirs, but only the cycling group demonstrated slight larger increased score in symptom subdomain (12.24 ± 7.95). Based on the MCID of the subdomain in KOOS (KOOS-symptoms: 5.1, KOOS-ADL: 17, and KOOS- QoL: 16.5) [33], the cycling group had a person demonstrated worse performance in symptoms and ADL subdomain clinically, and also one person in each ADL and QoL subdomain presented clinical improvement. In the EBF group, two people had clinical improvement in each symptom and QoL subdomain, and only one person reached the clinical difference in the ADL subdomain. No one in the EBF group showed more impairment in the functional questionnaire. As mentioned above, our six-week cycling training had similar functional improvement with twelve-week training, and that may reflect that six-week training is enough for improving OA knee patients’ function.

After six weeks of training, both groups demonstrated a few enhancements in muscle strength, and the isometric strength in the contralateral side in the EBF group decreased. Also, there were no significant differences between groups regarding the change in muscle strength (Table 3). The result of no significant difference between with and without EBF was also shown in a previous study using EBF for quadriceps strengthening, and the training load was progressively increased from 0 to 1.5 kg [24]. Although the EBF group’s contralateral side demonstrated decreased muscle strength, the amount of this change was small, and there were nearly no changes. Salacinski et al. (2012) designed a cycling training program at 70–75% of maximal heart rate with two sessions per week for twelve weeks, and their results also showed no significant increase in muscle strength after training [21]. An exercise program with at least 1 h, 3 to 5 sessions per week for 8–12 weeks was recommended to get a significant training effect in knee OA patients [37]. A previous study suggested the loading of strengthening for healthy older females should be 40% of 2RM [38]. In our study, low-loading and shorter period was performed on the stationary bike, and this setup may limit the effect of training on muscle strength. So, the reason for the lack of improvement in muscle strength in our training program should be due to the shorter training duration and lighter loading. Therefore, increasing workload, longer training duration, and higher training frequency may be needed instead of the current six-week training program. The endurance, i.e., work fatigue, had significant side effects in our study, and the contralateral side presented more improvement than the affected side, esp. in the EBF group (Table 3). Asymmetrical performance between limbs during cycling exercises in OA patients was mentioned, and their results found that the affected side will produce more power [39]. Considering training with EBF, a previous study identified that EBF training could be more beneficial for endurance than loading feedback [40]. Although there was no significant difference between groups in our study, the EBF groups showed less increase and decreased work fatigue; less work fatigue means better endurance. According to the results of our study, the six-week cycling training could generate meaningful limited changes in muscle strength, and training with EBF may be beneficial for enhancing muscle endurance.

As in Table 4, few group main effects were identified among muscles, and the effect durations were inconsistent. During the whole cycling exercise, EBF training can enhance the VM and VL recruitment in the mid-term (VM: 20 min, VL: 15–25 min), and RF in the terminal (the last 5 min). These changes may come from increasing the average firing rate and the number of motor unit recruitment by changing the neuromuscular control [41]. Further, this result may support that our EBF training can influence neuromuscular control. This effect is present in the feedback muscle (VM) and other quadricep muscles. The contralateral side’s VL presented larger increases in muscle recruitment in both groups (side main effect), except for the last 5 min. A previous study also identified the asymmetrical performance between limbs during cycling exercise in OA patients [39]. After training in RF and VL, the changes showed interaction during the whole exercise time and VM at the beginning and termination during the cycling exercise. In Fig. 3, we noted that the cycling group showed a slight increase or even decrease in RF in the maximum EMG amplitude, but all three muscles demonstrated an increase in the EBF group. This increment of VL muscle activation may correlate with pain relief [42]. Therefore, the EBF training seems to have some benefits in enhancing quadricep muscle recruitment in knee OA patients.

Because the VM plays an essential role in influencing pain intensity and knee function [43–46], enhancing the recruitment of VM was set to be the main target in our EMG biofeedback intervention. However, the effect of biofeedback in our study is not only present in the VM but also in the RF and VL. The maximum muscle activation of all muscles in the EBF group was more elevated than that of the cycling group. (Fig. 3).

### Limitations

This study had the following limitations. The first limitation was the small sample size, with only seven participants in each group. Therefore, this study focused on assessing the feasibility of EMG biofeedback training in patients with OA. The results suggest that incorporating biofeedback into the training program may offer greater benefits compared to training without biofeedback. The second limitation was the short training period. Knee OA is a chronic disease and progresses over time, so it may take more time to produce noticeable physiological and functional changes between groups.

## Conclusion

In summary, this six-week training program, with 30 min per session twice per week, could demonstrate some training effects in releasing pain and enhancing knee joint function, but there is almost no change in muscle strength. With the EMG biofeedback in VM, it could be used for alternating neuromuscular control of quadricep muscles during cycling and had a trend of helping to decrease the frequency of pain, enhance functional performance, muscle endurance, and increase muscle activation, especially in RF and VL. This study thus suggests that combining stationary cycling with EMG biofeedback training can enhance neuromuscular control of the vastus lateralis and may contribute to improved functional performance, especially by reducing pain in patients with osteoarthritis. Further studies with larger sample sizes are needed to confirm the representative effects of this six-week EMG biofeedback training program. Additionally, investigating the effects of different training dosages—such as increased loading or extended duration—may help identify the optimal protocol.

## Data Availability

No datasets were generated or analysed during the current study.

## Declarations

### Clinical trial registration

The protocol of this study was registered in ClinicalTrials.gov (NCT03484910) on 31st March, 2018.

## Abbreviations

ADL: Activities of daily living
BDC: Bottom dead center
BW: Body weight
EBF: Electromyographic biofeedback
EMG: Electromyographic
KOOS: Knee injury and osteoarthritis outcome score
MCID: Minimal clinically important difference
MVC: Maximum voluntary contraction
OA: Osteoarthritis
QoL: Quality of life
RF: Rectus femoris
RMS: Root mean square
RPM: Revolutions per minute
TQ: Torque
VAS: Visual analog scale
VL: Vastus lateralis
VM: Vastus medialis
